# Fabrication of biochar-based superhydrophobic coating on steel substrate and its UV resistance, anti-scaling, and corrosion resistance performance

**DOI:** 10.1038/s41598-023-36589-0

**Published:** 2023-06-10

**Authors:** M. E. Mohamed, O. Adel, E. Khamis

**Affiliations:** 1 Chemistry Department, Faculty of Science, Alexandria University, Alexandria, Egypt; 2 Faculty of Advanced Basic Sciences, Alamein International University, Alamein City, Matrouh Governorate Egypt; 3 Egyptian Russian University, Badr, Egypt

**Keywords:** Chemical engineering, Chemistry, Materials science

## Abstract

In this study, we report an eco-friendly and facile process for the synthesis of biochar, BC, and a cobalt-biochar nanocomposite, Co-BC, using rice straw biomass. We constructed two superhydrophobic coatings on steel substrates using potentiostatic electrodeposition of nickel-modified biochar, Ni@BC, and nickel modified by cobalt-biochar nanocomposite, Ni@Co-BC, then, these coatings were soaked in an ethanolic stearic acid solution. Fourier transform infrared spectroscopy showed that the stearic acid-grafted Ni@BC coating, Ni@BC@SA, and the stearic acid-grafted Ni@Co-BC composite, Ni@Co-BC@SA, were well grafted on the steel surface. Scanning electron microscopy revealed that the superhydrophobic coatings have nanoscale features. Atomic force microscopy results showed that the Ni@Co-BC@SA coat had higher roughness than Ni@BC@SA, resulting in higher superhydrophobicity. The water contact angles for Ni@BC@SA and Ni@Co-BC@SA coatings were 161° and 165°, respectively, while the values of water sliding angles for both coatings were 3.0° and 1.0°, respectively. Quantitative estimation of the scale inhibition efficiency revealed that the Ni@Co-BC@SA coating exhibited greater efficiency compared to the Ni@BC@SA coating. Additionally, the Ni@Co-BC@SA coating demonstrated improved corrosion resistance, UV resistance, mechanical abrasion resistance, and chemical stability compared to the Ni@BC@SA coating. These results highlight the superior performance of the Ni@Co-BC@SA coating and its potential as a highly effective and durable superhydrophobic coating for steel substrates.

## Introduction

Wide-ranging industrial applications are anticipated for several synthetic superhydrophobic, SHP, surfaces that were inspired by lotus leaves^1^. SHP surfaces are exceptionally water-repellent surfaces with a water contact angle, WCA, of more than 150° and a water sliding angle, WSA, of lower than 10°^2,3^. Due to the significance of SHP surfaces in both fundamental research and practical applications, they have received a lot of attention. It is common knowledge that surface-wetting behaviour is determined by the combination of rough surfaces and various surface energies. Low surface energy rough surfaces are typically SHP, whereas high surface energy rough surfaces are typically superhydrophilic^4^. Perfluorinated compounds, such as fluoro silanes or fluorocarbon molecules, have historically been used as low surface energy materials due to their exceptionally low surface energy^4,5^. However, it has been demonstrated that employing such long-chained fluorocarbons has very harmful side effects, including persistence, biomagnification, and bioaccumulation^5–9^. So, it can be challenging to design a SHP surface with these characteristics, particularly when there are concerns about environmental safety. As a result, it's essential to develop low-cost, environmentally friendly procedures and materials for producing SHP surfaces^5,10^.

SHP surfaces have a broad array of uses, including corrosion resistance, UV resistance, oil–water separation technologies, etc.^11–18^. Several techniques have been presented for the development of SHP coatings, including electrodeposition, electrochemical anodic oxidation, anodization, etc.^19–26^. Due to its simplicity, low-temperature procedure, clean, affordable, and adjustable nanostructure, electrodeposition is a great method for designing artificial SHP surfaces^3^. Because of its inexpensive cost and superior mechanical properties, carbon steel is the construction material that is most frequently utilized in numerous industries. It is employed in huge quantities in equipment for metal processing, building, bridges, chemical processing, petroleum production, and marine applications^27,28^. Steel corrosion and its suppression under these conditions are complicated process problems. Corrosion is typically viewed as one of our society’s most critical problems, with economic and safety ramifications^29–31^. Steel surfaces can be protected using a variety of methods, the development of SHP coatings, which significantly increase steel corrosion resistance, is one of the most crucial^32,33^.

SHP surfaces can be used in practical applications, but their mechanical instability restricts their use^34,35^. SHP coatings need to have higher chemical stability and mechanical abrasion resistance in order to be employed in industrial applications.

Biochar, BC, is a porous carbonaceous substance, and it is created once a biomass feedstock, for example, wood chips, manures, seed leftovers, and rice straw, is pyrolyzed in a restricted quantity of oxygen^36^. Biochar has increasingly become more popular in recent years. Biochar has a tremendous potential to replace graphene in various applications since it is less expensive than graphene (0.25 USD/kg for biochar vs. 1400 USD/kg for graphene)^37^. Around the world, BC is utilized as an efficient adsorbent to eliminate various types of contaminants in water^38^. The surface area of BC is increased by modification with metallic nanoparticles such as cobalt and nickel^39^. Cobalt, Co, is frequently employed in the fields of magnetic recording, aerospace, shipbuilding, corrosion resistance, and high-strength alloys^40,41^. These many qualities are determined by the material's shape and internal properties^42,43^. Controlling the emergence of distinctive cobalt nanostructures has consequently emerged as a crucial problem in the materials fabrication sector. To our best knowledge, it is the first report for the construction of SHP coatings based on BC and biochar modified by cobalt, Co-BC, that could be used as UV, anti-scaling, and corrosion-resistant materials. In this study, we use BC and Co-BC as an additive to enhance the surface roughness, this is the primary condition to achieve SHP characteristics. Among all agricultural products, rice straw is the greatest commonly used with 120 million tons produced annually^44^. Recently, the majority of farmers have chosen to burn rice straw as it is the most straightforward way of production. However, if the number of burnings rises, this has serious adverse impacts, such as air pollution. This negative environmental impact is minimized by transforming this waste into more desirable materials such as BC.

This research attempts to construct a BC and Co-BC based SHP coating on the carbon steel (ASTM A283/Grade C) surface. ASTM A283/Grade C steel commonly used in the construction industry, pressure vessels, towers, tanks, automotive industry, the railroad cars and structural applications of medium strength requirement^45–48^. Low-cost and environmentally safe stearic acid is utilized as a low surface energy compound^49^. Biochar was synthesized by an environmentally eco-friendly method from the rice straw. The wettability, chemical and mechanical stability, UV resistance, anti-scaling, and corrosion performance were assessed for the prepared SHP coatings.

## Experimental

### Materials

As a substrate, a steel plate (ASTM A283/Grade C) with the following measurements: 2.0 cm, 1.0 cm, and 0.1 cm was employed. The rice straw was collected in accordance with institutional, national, and international guidelines and legislation. Nickel sulfate, nickel chloride hexahydrate, anhydrous ethanol, cobalt sulfate heptahydrate, boric acid, sulfuric acid, sodium chloride, stearic acid, and sodium hydroxide of analytical grade were used.

### Biochar synthesis from rice straw

The process for manufacturing BC involved thoroughly washing the rice straw to get rid of any impurities, then drying it in the air before putting it in an oven for 24 h at 60 °C. The clean, dried rice straw was then processed via a mixer to create a fine powder. The BC was then produced by pyrolyzing ten grams of the fine powder for three hours at 600 °C in a muffle furnace.

CoSO4⋅7H_2_O (4.8 g) was added to 100 mL of deionized water containing 10 g of the rice straw fine powder with a wt% ratio of 1:10 for Co: rice straw fine powder. The mixture was sonicated for 30 min and then stirred for 1.0 h. After that, the mixture was oven-dried at 60 °C overnight. After that, it was pyrolyzed in a muffle furnace at 600 °C for three hours to obtain the cobalt-modified biochar material, Co-BC.

### Superhydrophobic coating construction

Before electrodeposition, emery paper of various classes was used to mechanically polish the steel substrate, starting with coarse (grade 120) and working up to the finest (800 grade). After being degreased in a solution of soap for ten minutes, the substrate was then immersed in 2.0 M H_2_SO_4_ for one minute, washed with distilled water, and then put straight into the electrodeposition bath. Table 1 shows the electrodeposition considerations for creating a Ni@BC and Ni@Co-BC coating on the steel substrate. The platinum sheet was utilized as the anode and was placed apart from the steel substrate, acting as the cathode, by a spacing of 2.0 cm. Ni@BC and Ni@Co-BC coatings that had been made were washed with distilled water and allowed to dry at room temperature overnight. Then, the substrates were placed in ethanolic solutions containing 1 × 10^–2^ M stearic acid, SA, for 15 min before being dried at room condition for 24 h. Different characterization and evaluation procedures were applied to the Ni@BC coating modified with stearic acid, Ni@BC@SA, and the Ni@Co-BC coating modified with stearic acid. Ni@Co-BC@SA.

**Table 1:** Bath constituents and working environments for electrodeposition of Ni@BC@SA and Ni@Co-BC@SA coating on the steel surface.

Factor		Level
(Source of nickel ion)	NiCl_2_⋅6H_2_O	40 g/L
	NiSO_4_	176 g/L
(Buffer the pH)	H_3_BO_3_	60 g/L
SLS		0. 4 g/L
BC or Co-BC		0. 4 g/L
Time of deposition		6.0 min
Deposition potential		11.0 V

### Surface characterization

Utilizing a Fourier transform infrared spectrophotometer, FTIR, the surface’s chemical composition was examined (model: Bruker Tensor 37 FTIR). An X-ray diffractometer was used to conduct an X-ray diffraction analysis using monochromatic Cu K radiation (Bruker D2 phaser). The surface topography of the created SHP coatings was examined utilizing a scanning electron microscope, SEM (model JSM-200 IT, JEOL). The atomic force microscopy, AFM, was accomplished by Scanning Probe Microscope (SPM9600—Shimadzu Japan). Utilizing an optical contact angle goniometer, the WCA and WSA were calculated using 5 µL water droplets (Rame-hart CA instrument, model 190-F2). The WCAs and WSAs values that are displayed are the averages of three measurements made at various substrate locations.

### Chemical stability

After being submerged in solutions with different pH values (pH 1–13) for 30 min, the produced SHP films were tested for both WCA and WSA at each pH^50,51^. In order to study how prolonged immersion affects the superhydrophobicity of a coating, we examined the coating’s performance under varying pH levels (3, 7, and 11) for immersion periods of 0.5, 2, 4, and 6 h. The pH value of the solution was altered using sodium hydroxide and sulfuric acid.

### Mechanical abrasion

The scratch test was used to evaluate the SHP coatings mechanical abrasion characteristics. 5.0 kPa pressure was affected on the samples of the created SHP coating that were set up on 800 mesh sandpaper. The WCA and WSA were measured for each 10.0 cm of horizontal movement of the produced SHP sample. The mechanical abrasion resistance that has been presented is an average of measurements made on two distinct samples.

### Corrosion test

An ACM frequency response analyzer and a three-electrode cell were used for the electrochemical measurements (UK). The counter electrode was a graphite rod, while the reference electrode was an Ag/AgCl electrode. Working electrodes comprised bare and covered steel with SHP Ni@BC@SA and Ni@Co-BC@SA films. The working electrodes were covered with an epoxy coating, leaving 1 cm^2^ open to the test solution. The working electrode was inserted into a 0.5 M NaCl solution-filled cell for 25 min at room temperature before electrochemical tests to achieve the rest potential. Electrochemical impedance spectroscopy, EIS, observations had a frequency range of 0.01 ≤ f ≤ 1.0 × 10^4^ and a signal amplitude of 10 mV around the open circuit potential. At a scan rate of 30 mV/min the potentiodynamic polarization, PDP, measurements were made with a potential range of − 250 to + 400 mV around the open circuit potential. To make sure that the measurements were precise, experiments were double-checked, and the outcomes were correct within 2% error.

### Scaling test

The anti-scaling performance was evaluated by weighting different samples of the uncoated and prepared SHP coated-steel samples and then soaking them in a solution of 0.01 M NaHCO_3_ and 0.01 M CaCl_2_ at 60 °C for a time ranging from 2 to 20 h. The samples were dried at room condition and reweighted. The weight gain, and difference in weight before and after immersion in 0.01 M NaHCO_3_ and 0.01 M CaCl_2_ were measured, which is equivalent to the rate of scale formed at the substrate surface. The weight gain is caused by CaCO_3_ deposition on the samples.

### UV resistance tests

The wettability of the prepared SHP surface at various time intervals under UV irradiation (λ = 365 nm, 300 W) was used to test its UV resistance. Every two hours, the WCA values were determined. The UV lamp and SHP coating remain spaced apart by about 10 cm. By analyzing the same sample at five separate sites, the average values were calculated.

## Results and discussion

### Composition of the fabricated coatings

**Figure 1:**
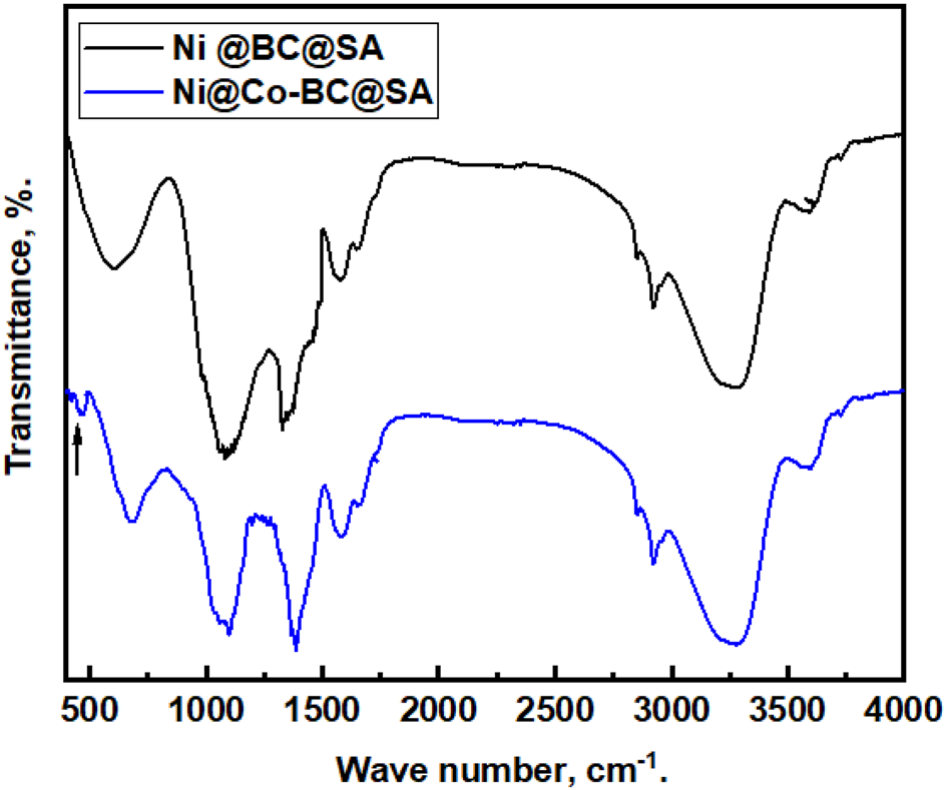
FTIR spectra of coated steel by Ni @BC@SA and Ni@Co-BC@SA.

The Fourier transform infrared spectrophotometer, FTIR, was used to analyze the surface’s chemical composition of the fabricated coats. Figure 1 displays the FTIR spectra of coated steel with Ni@BC@SA and Ni@Co-BC@SA. The spectra of steel processed with Ni@BC@SA has large absorption peaks at 3278 cm^−1^, which are the stretching vibration modes of the –OH group^52^. The asymmetry and symmetry vibration of the stearic acid’s –CH_2_– is attributed to the peaks at 2856 cm^−1^ and 2922 cm^−1^, respectively^53^. The shoulder near 1579 cm^−1^ matched the stretching vibration of C=O, and C=C^36^. The O–H bending or C–O stretching vibration of phenol is responsible for the peak at 1342 cm^−1^^38^. The bending vibration of –C–OH is responsible for the peak at 1083 cm^−1^^38^. Ni(OH)_2_ corresponds to the peak at 716 cm^−1^^32^. The spectra of steel coated with Ni@Co-BC@SA exhibit the same peaks as those of Ni@BC@SA with an additional peak at 471 cm^−1^, which is attributed to Co_3_O_4_^54,55^.

**Figure 2:**
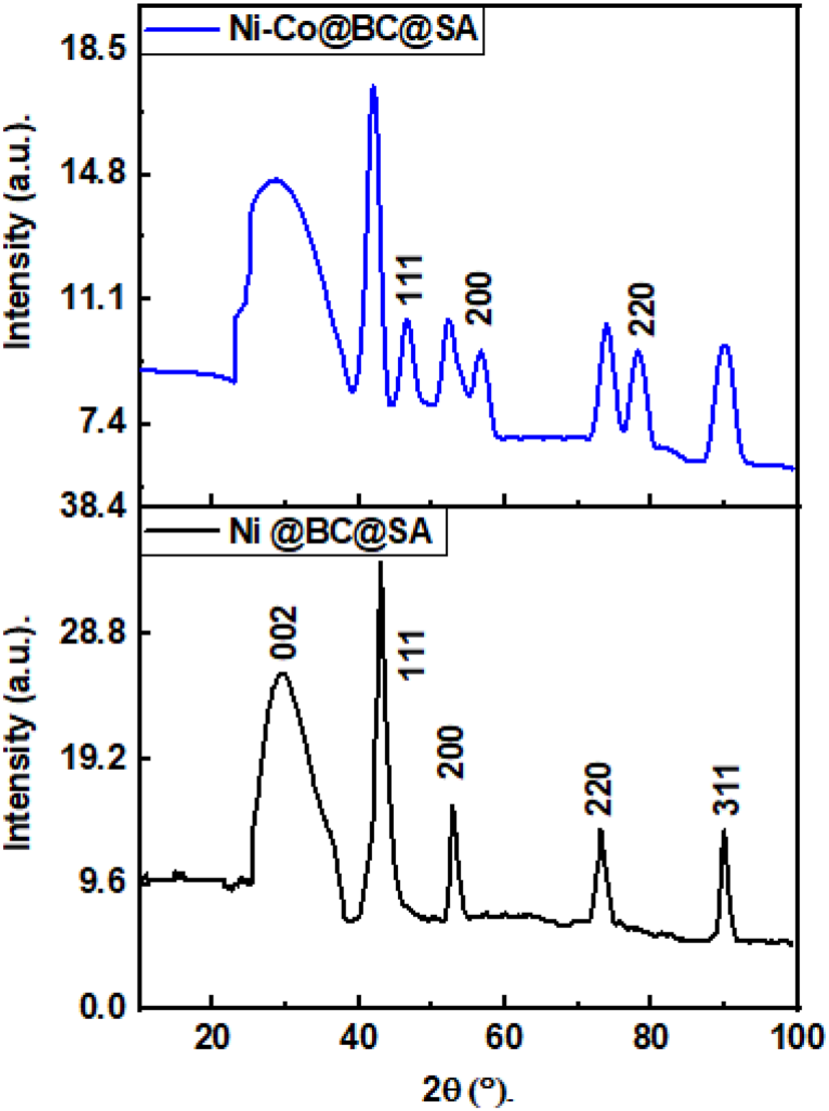
XRD patterns of the SHP coated steel with Ni @BC@SA and Ni@Co-BC@SA.

The XRD technique was used to determine the crystal orientation and composition of steel grafted by Ni@BC@SA and Ni@Co-BC@SA SHP coatings. Figure 2 displays the XRD patterns of various prepared coatings. The Ni @BC@SA coat exhibits 5 diffraction peaks in its XRD pattern. The four peaks at 2Ɵ values equal 42.8°, 53.1°, 73.3°, and 89.9° are related to the faced cubic centered, fcc, of Ni (JCPDS NO. 04-0831). The XRD peak at 2Ɵ values equals 28.9° is corresponds to biochar^56,57^.

While the XRD pattern of the Ni@Co-BC@SA composite demonstrated the same peaks of the Ni @BC@SA with a lower intensity and other new peaks at 46.2°, 56.4°, and 78.5° which are referring to face-centered-cubic cobalt^58^. The XRD peaks of steel coated with Ni@Co-BC@SA are broad, showing that the deposited structures have small-sized particles.

### Morphology and wettability of the fabricated coatings

**Figure 3:**
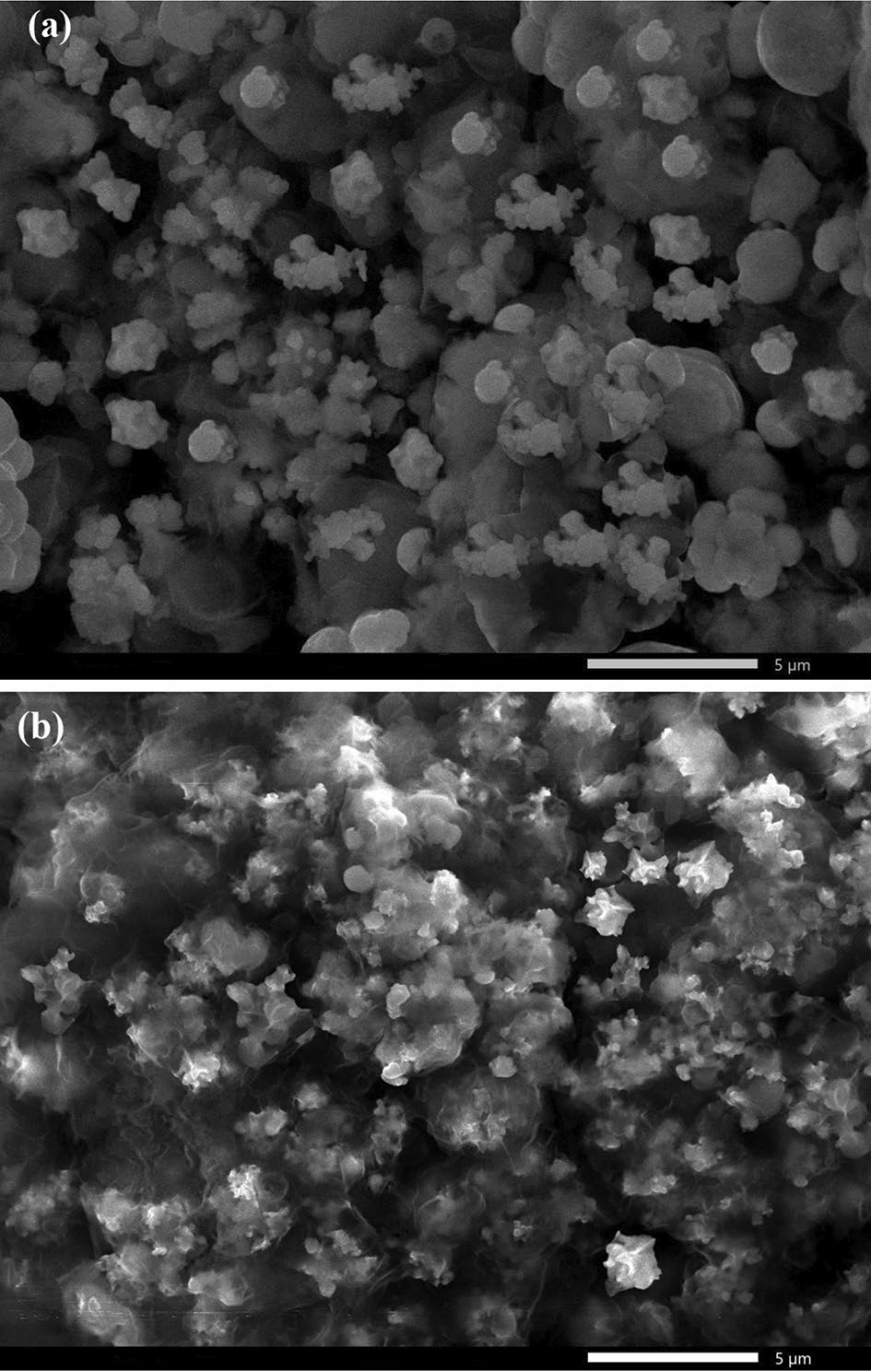
SEM micrographs of the SHP coated steel with (**a**) Ni @BC@SA and (**b**) Ni@Co-BC@SA.

One of the most crucial things to take into account while analyzing SHP features is surface morphology, so the topography of the produced SHP coatings on the steel substrate has been investigated using the SEM technique. A micrograph of coated steel with Ni@BC@SA is shown in Fig. 3a, it is obvious that the formed structures contain particles with a diameter of only a few nanometers. Some of the nanoparticles produce larger aggregated particles. Figure 3b shows a micrograph of steel that has been grafted with Ni@Co-BC@SA film. The Figure demonstrates that the deposited structures contain smaller, circular-like nanoparticles than Ni@BC@SA film. Apparently, the Co might act as a nucleation site and speed up the nucleation process rather than crystal growth, which is why the Ni@Co-BC@SA coating contains smaller nanoparticles^59,60^. The Ni@Co-BC@SA hence exhibits stronger superhydrophobicity due to its higher surface roughness. The transparent flakes of BC layers are clearly seen especially in the case of Ni@Co-BC@SA.

The WCAs and WSAs were measured to ascertain the wettability behavior of the constructed SHP coatings. Ni@BC@SA and Ni@Co-BC@SA films have WCA values of 161° and 165°, respectively, while both films have WSA values of 3.0° and 1.0°. These findings suggest that Co promotes superhydrophobicity and roughness. The air that can be stored in the nanostructures effectively prevents water from touching the surface^61^. Additionally, Ni@Co-BC@SA coated steel has wettability that is advanced to numerous earlier documented values^62–65^.

### Roughness of the fabricated coatings

**Figure 4:**
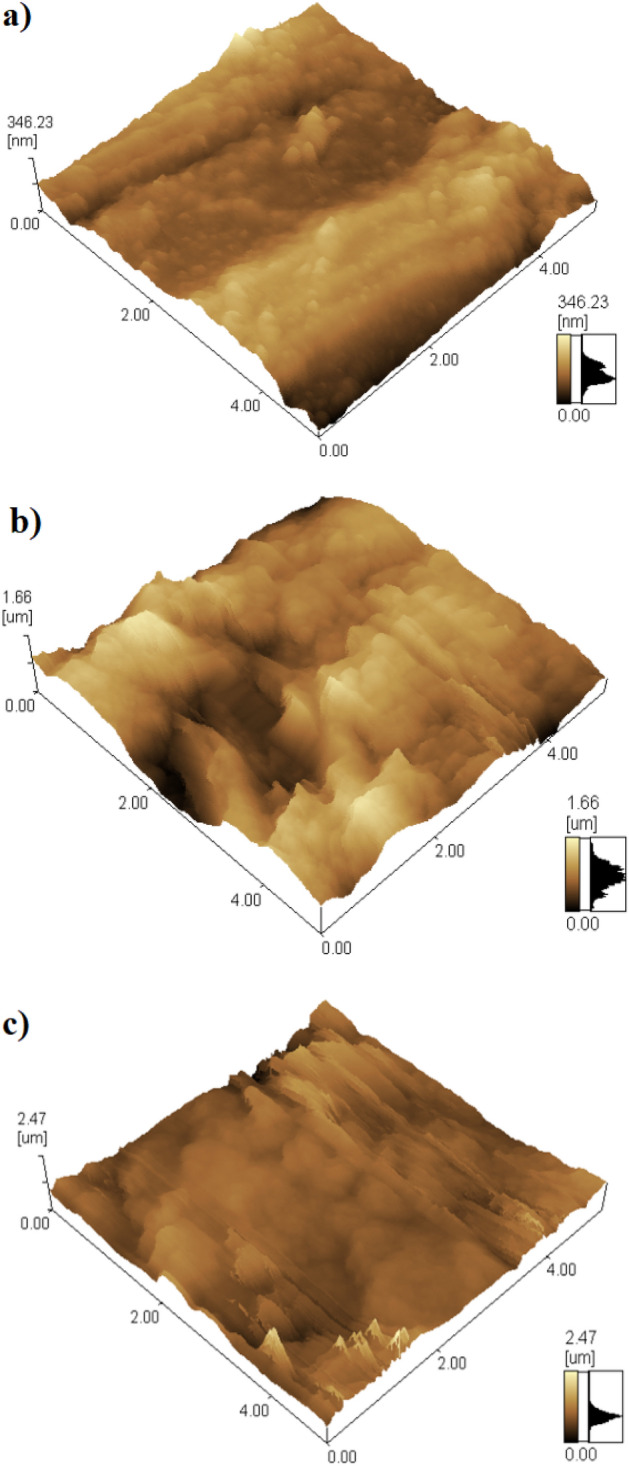
3D AFM topography images of the surface of (**a**) bare and SHP coated steel with (**b**) Ni @BC@SA and (**c**) Ni@Co-BC@SA.

The surface roughness of the bare and SHP coated steel was further characterized using the AFM. According to the 3D AFM image, Fig. 4a, the bare steel’s arithmetic average roughness, Ra, was 0.34 µm. For SHP coated steel by Ni@BC@SA, the Ra value increased to 1.60 µm, showing that the deposited coat increases the steel surface roughness, Fig. 4b. Figure 4c shows that the Ra value for SHP coated steel by Ni@Co-BC@SA increased to 2.21 µm, which can be attributed to the BC doping with cobalt, which significantly increases the roughness of the steel surface.

### Chemical stability

**Figure 5:**
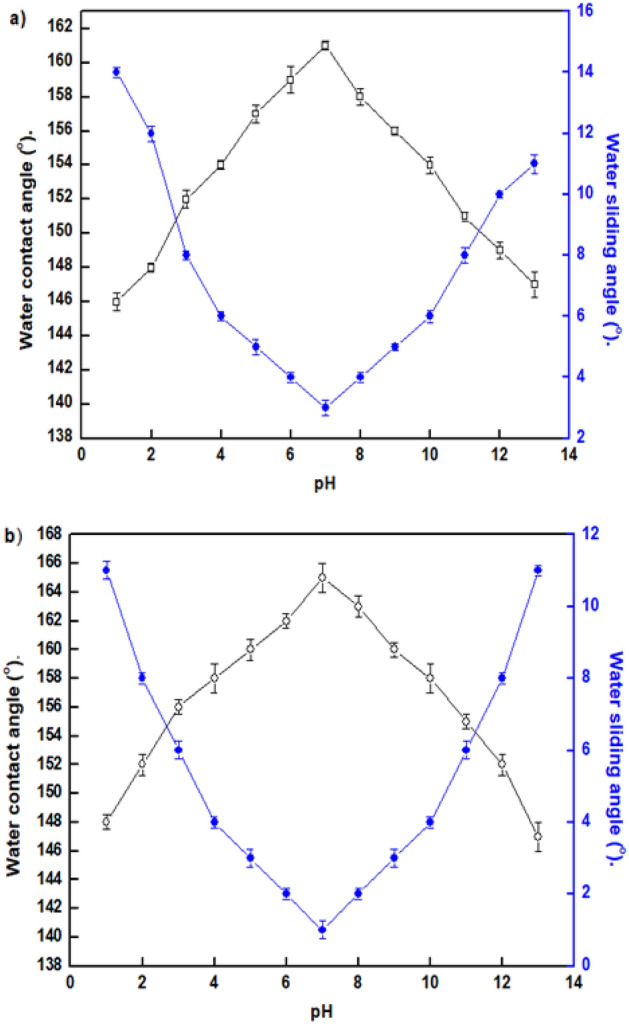
The change in solution pH with the water contact angle and water sliding angle of the coated steel by (**a**) Ni@BC@SA, and (**b**) Ni@Co-BC@SA.

**Figure 6:**
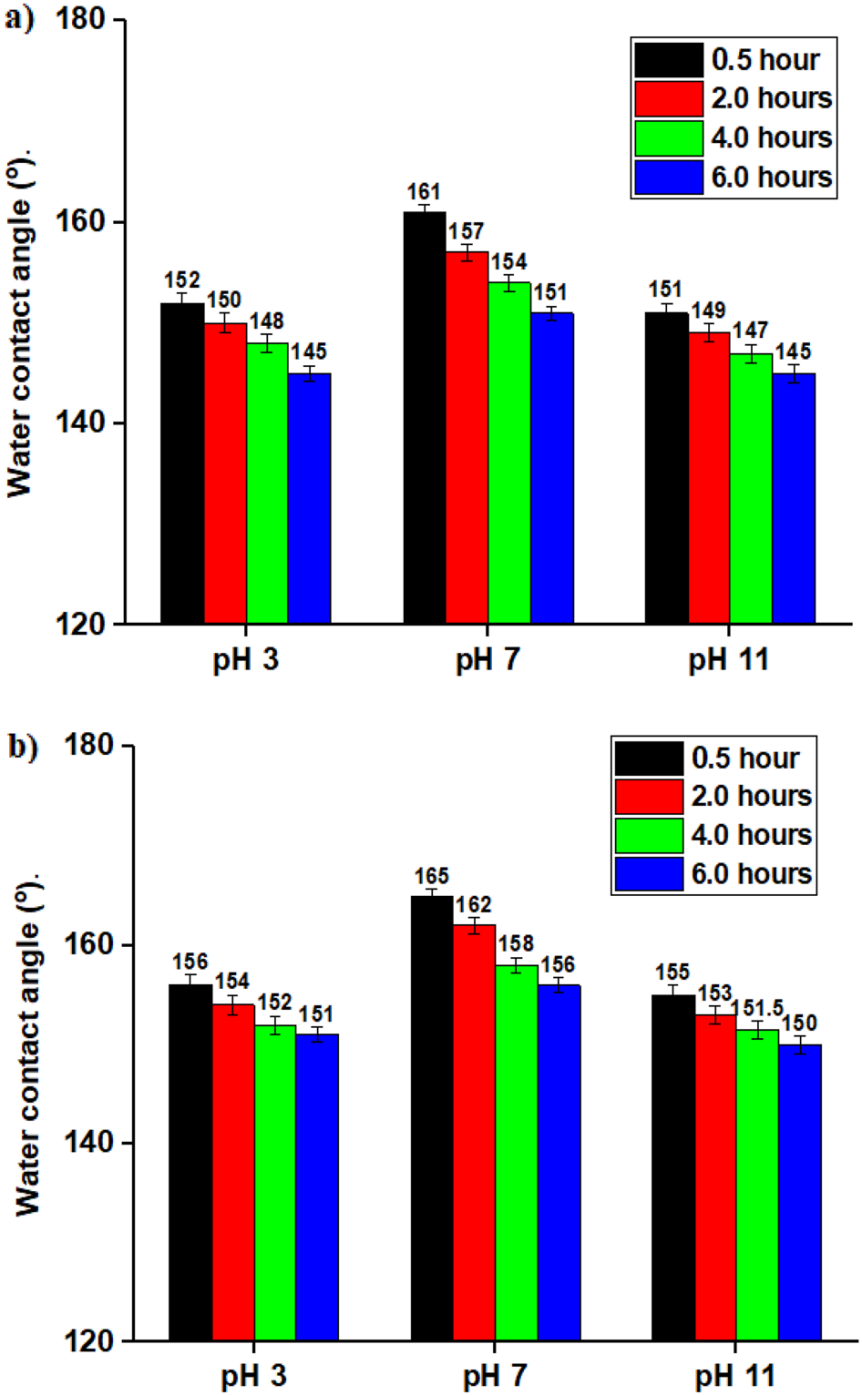
The change in solution pH with the water contact angle at different immersion time of coated steel by (**a**) Ni@BC@SA, and (**b**) Ni@Co-BC@SA.

To demonstrate that the created SHP film may be utilized in the industrial sector, a chemical stability test must be carried out. The correlations between pH and the WCAs and WSAs of water droplets on the produced SHP coatings are displayed in Fig. 5. Figure 5a shows that the Ni@BC@SA films are SHP between pH values of 3 and 11, while Fig. 5b shows that the Ni@Co-BC@SA films are SHP between pH values of 2 and 12, where the WCAs are frequently greater than 150° and the WSAs are less than 10°. As a result, incorporating Co to BC enhances the SHP coating’s chemical stability in both basic and acidic environments. To investigate the effect of prolonged immersion time of the coating on its superhydrophobicity, we measure the WCA of a coating in different pH values (3, 7, and 11) at different immersion times 0.5, 2, 4, and 6 h, Fig. 6. The results show that, at pH 7 the coated steel with Ni@BC@SA retains its superhydrophobic characteristics at all examined immersion periods where the WCA is always greater than 150°, while for pH 3, the coat retain superhydrophobicity until immersion time of 2 h, and finally for pH 11, the coat retains superhydrophobicity until immersion time of 0.5 h. While the coated steel with Ni@Co-BC@SA retains its superhydrophobic characteristics at the different pH until immersion time of 6 h (the maximum examined immersion time). The SHP coated steel with Ni@Co-BC@SA has greater chemical stability than several previously known values^62,66^.

### Mechanical stability

**Figure 7:**
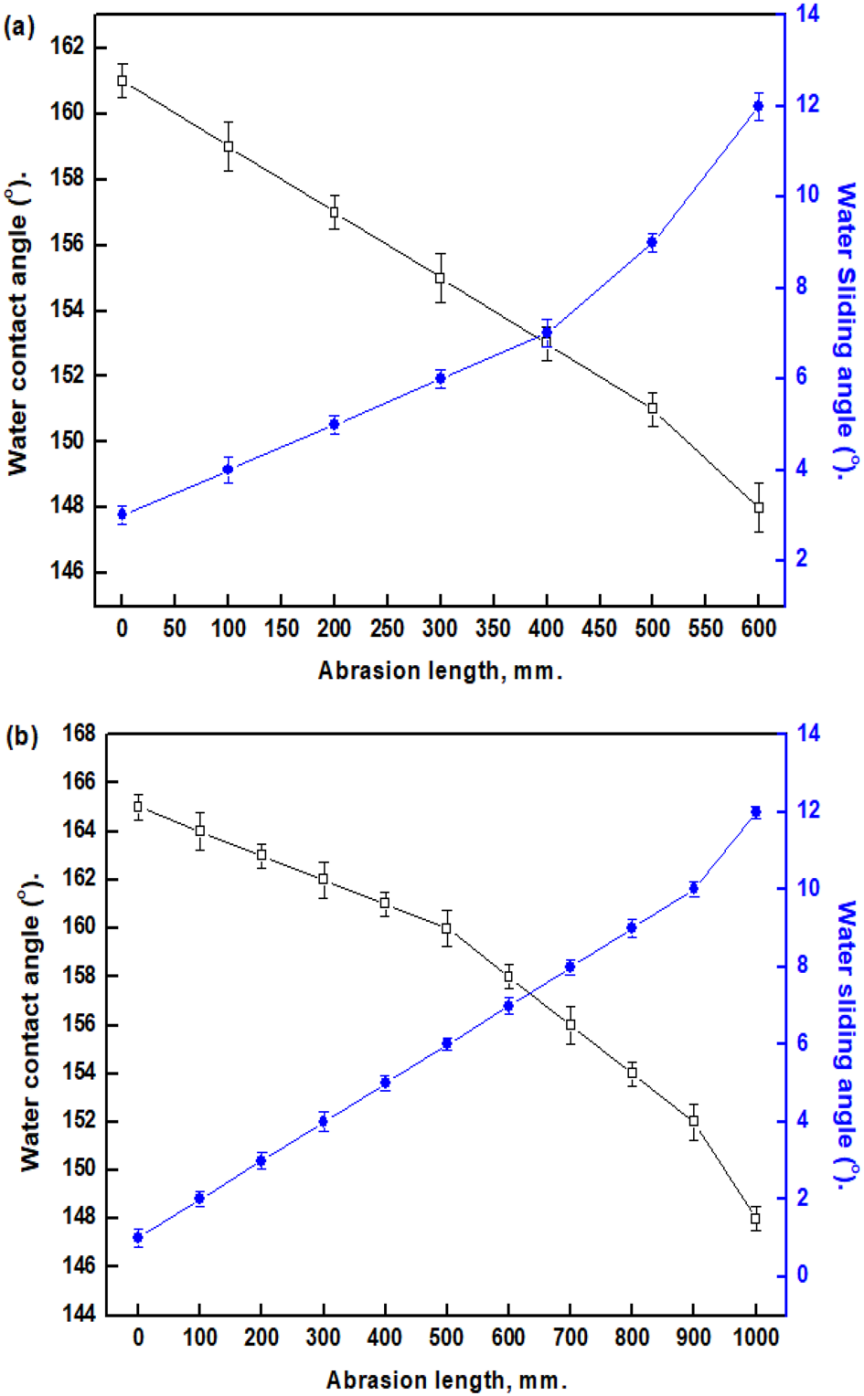
Change of WCAs and WSAs with the length of abrasion for coated steel with (**a**) Ni @BC@SA and (**b**) Ni@Co-BC@SA.

Mechanical abrasion can damage the SHP surfaces. Even when touched with the finger, some SHP surfaces can crack^67^. The primary focus now is on enhancing the abrasion resistance of SHP coatings so can use in the industrial sector^68^. The prepared SHP films were subjected to abrasion tests to determine their resistance to mechanical abrasion. Figure 7 depicts how the abrasion length affects the changes in WCAs and WSAs of the prepared SHP films.

The prepared Ni@BC@SA SHP film retains its SHP property until a 500 mm abrasion length. While the prepared Ni@Co-BC@SA SHP film retains its SHP property until a 900 mm abrasion length. These findings showed that adding Co to the developed SHP BC-based film greatly increased its mechanical stability. The coated steel with Ni@Co-BC@SA has greater abrasion resistance than several previously known values^69–72^.

The durability of the SHP sample is investigated via storage in an ambient atmosphere. After three months of storage in air, the values of the WCAs of Ni@BC@SA and Ni@Co-BC@SA films are 151° and 158°, and the WSAs are 9° and 5°, respectively. These findings show that the produced SHP films demonstrate long-term stability and durability and are stable in air.

### Corrosion resistance behaviour

#### PDP results

**Figure 8:**
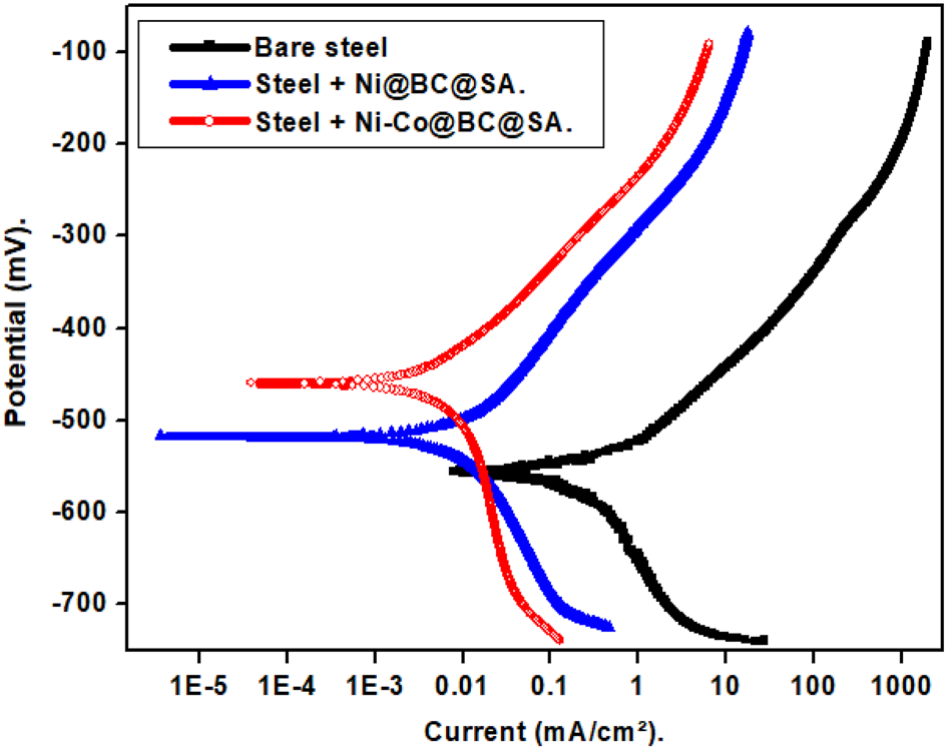
The PDP plots for the bare and the SHP coated-steel in 0.5 M NaCl solution.

The PDP technique has been used to examine the corrosion behavior of bare and SHP coated steel by Ni@BC@SA and Ni@Co-BC@SA. Figure 8 displays the PDP plots of uncoated and SHP coated steel in a 0.5 M NaCl aqueous solution. The oxygen reduction reaction is represented by limiting diffusion currents in the cathodic polarization curves, Eq. (1)^73^.

1 $${\text{O}}_{{2}} + {\text{2H}}_{{2}} {\text{O }} + {\text{ 4e}} \to {\text{4OH}}^{ - } .$$

Therefore, the cathodic process is controlled by the oxygen gas diffusion from the bulk to the surface of the electrode. The development of an ideal anodic Tafel area is hindered by the rapid generation of corrosion products on the electrode surface for the bare steel, or by the development of a passive layer when the steel is treated with a SHP coating^74,75^.

The PDP parameters including corrosion potential (E_corr_), corrosion current density (i_corr_), and protection efficiency (%P) of bare and SHP coated steel are displayed in Table 2. The protection efficiency was calculated using Eq. (2)^76^.

2 $$\% {\text{P}} = \left[ {\left( {{\text{i}}_{{{\text{o}}.}} - {\text{i}}} \right)/{\text{i}}_{{{\text{o}}.}} } \right] \times { 1}00,$$

where, i_o._ and i are the corrosion current density of the bare and SHP coated steel. Because of the coated steel’s SHP properties, the i_corr_. value for coated steel with Ni @BC@SA is smaller than that for bare steel. The SHP coating microstructures’ trapped air can lower the contact area between the steel and solution, which causes a more rapid decrease in the i_corr_ value^77^. The doping of biochar with cobalt enhances the SHP coating property and leads to a higher reduction in the contact area of the medium and steel. Therefore, steel coated with Ni@Co-BC@SA has a greater protection efficiency than coated steel with Ni @BC@SA.

**Table 2:** The PDP parameters for the bare and the SHP coated-steel in 0.5 M NaCl solution.

Deposit	− E_corr_ mV	β_a_ mV/decade	− β_c_ mV/decade	i_corr_ µA/cm^2^	%P
Bare steel	672.10	106.34	144.62	26.29	–
Steel + Ni@BC@SA	526.52	129.05	159.81	2.22	91.56
Steel + Ni@Co-BC@SA	429.13	82.229	386.89	1.12	95.74

#### EIS results

**Figure 9:**
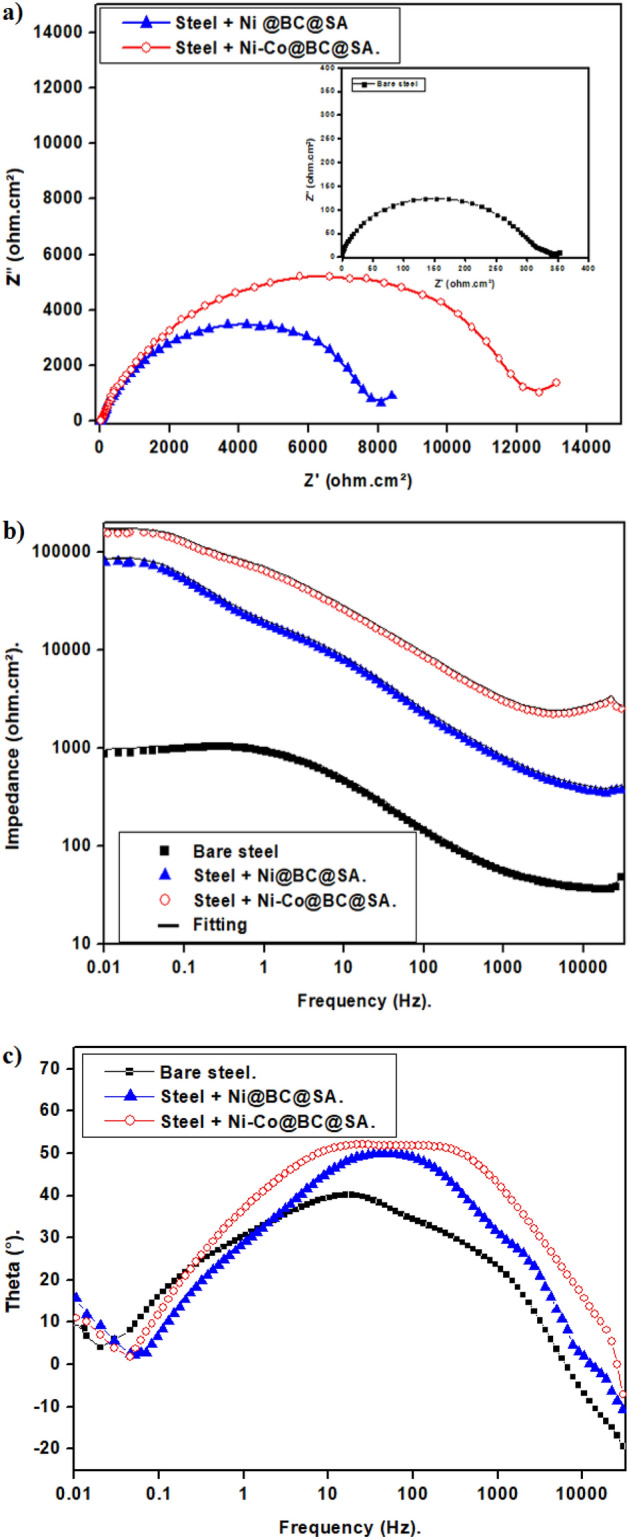
(**a**) Nyquist, (**b**) Bode and (**c**) Theta plots of bare and SHP coated steel in 0.5 M NaCl solution.

Figure 9 displays the Nyquist and Bode plots of uncoated and SHP coated steel in a 0.5 M NaCl solution. The Nyquist plots, Fig. 9a, exhibit a depressed capacitive semicircle at high frequency and a diffusion tail at low frequency. The interfacial charge transfer reaction is what causes the depressed capacitive semicircle of the Nyquist plots at high frequencies^78,79^. Mass transport is responsible for the diffusion tail at low frequencies. These results indicate that the existence of a protective SHP layer is the reason why the steel coated with Ni@BC@SA exhibits superior charge transfer resistance than bare steel. The coated steel with Ni@Co-BC@SA displays the highest capacitive semicircle, indicating that it offers the greatest degree of protection. The doping of biochar with cobalt enhances the superhydrophobicity and so the Ni@Co-BC@SA coat becomes more effective to restrict the transfer of corrosive species like Cl^-^ and H_2_O into the surface of the steel metal.

The manufactured SHP coated steel in 0.5 M NaCl solution exhibits greater impedance at the low frequency on the Bode plots than bare steel, as shown in Fig. 9b. This confirms that the steel substrate is being protected by the created SHP coatings. Two-time constants are shown at low and intermediate frequencies in the phase angle plot, Fig. 9c. The unprotective corrosion products of bare steel or the protective SHP coating were responsible for the time constant that appeared in the low-frequency region. The electrical double layer was responsible for the time constant that appeared at the moderate frequency^80–82^.

**Figure 10:**
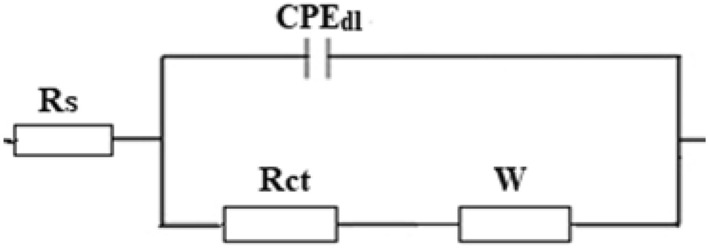
The equivalent circuit model.

The equivalent circuit depicted in Fig. 10 was employed to fit the EIS experimental results and the Zsimpwin program was used to determine the impedance parameters. The components of the equivalent circuit are; charge transfer resistance, R_ct_, double-layer constant phase element, CPE_dl_, solution resistance, R_s_, and Warburg element. W. Equation (3) was used to determine the protection efficiency^76^:

3 $$\% {\text{P}} = \, \left[ {\left( {{\text{R}}_{{{\text{ct}}}} - {\text{R}}_{{{\text{ct}}}}^{{\text{o}}} } \right)/{\text{ R}}_{{{\text{ct}}}} } \right] \, \times {1}00.$$

R_ct_^o^ and R_ct_ are the charge transfer resistance for the bare and SHP coated steel. The attained impedance parameters are displayed in Table 3. As it is obvious, each of Rct and %P of the bare steel < steel + Ni@BC@SA < steel + Ni@Co-BC@SA, and so the corrosion resistance increases in the same order. The corrosion resistance of the SHP coated steel by Ni@Co-BC@SA is greater than numerous formerly recorded values^83–85^.

**Table 3:** The EIS parameters for the bare and SHP coated steel in 0.5 M NaCl solution.

Deposit	Rs (Ohm cm^2^)	n_1_	CPE_dl_ × 10^–6^ (s^n^ Ω^−1^ cm^2^)	W × 10^–4^	R_ct_ (Ohm cm^2^)	%P
Bare steel	1.8	0.76	288.4	401.2	332	–
Steel + Ni@BC@SA	3.4	0.78	44.3	16.3	7978	95.84
Steel + Ni@Co-BC@SA	4.9	0.79	25.8	12.8	12,044	97.24

#### Mechanism of anti-corrosion performance

On the bare steel surface, the water molecules can readily adsorb. Uncoated steel can also suffer severe corrosion from chloride ions adhering to the surface and forming [FeClOH]^−^. As a result, the corrosion process can simply be initiated when Cl^-^ ions and water come into contact with the metal surface^86^.

On the other hand, steel that has been coated with SHP films has a nanostructure that has hydrophobic material adsorbed to it. The holes between the peaks of the rough surface are easily filled with air. Due to the obstructive effect of trapped air, aggressive ion species in corrosive environments, such as Cl^−^, may rarely attack the underlying surface^10,86,87^. The SHP surface in neutral solutions was also shown to be negatively charged. According to reports, the presence of electronegative functional groups gives biochar a negative zeta potential value^88–90^. A biochar-based SHP surface’s negative charge led to a drop in the amount of Cl^−^ anion present close to a solid surface, increasing corrosion resistance^10^. It is also reported that the cobalt oxide nanoparticles have a negative zeta potential value^91–93^. So, the steel coated with Ni@Co-BC@SA has enhanced corrosion resistance than the SHP Ni@BC@SA coating.

### Anti-scaling performance

**Figure 11:**
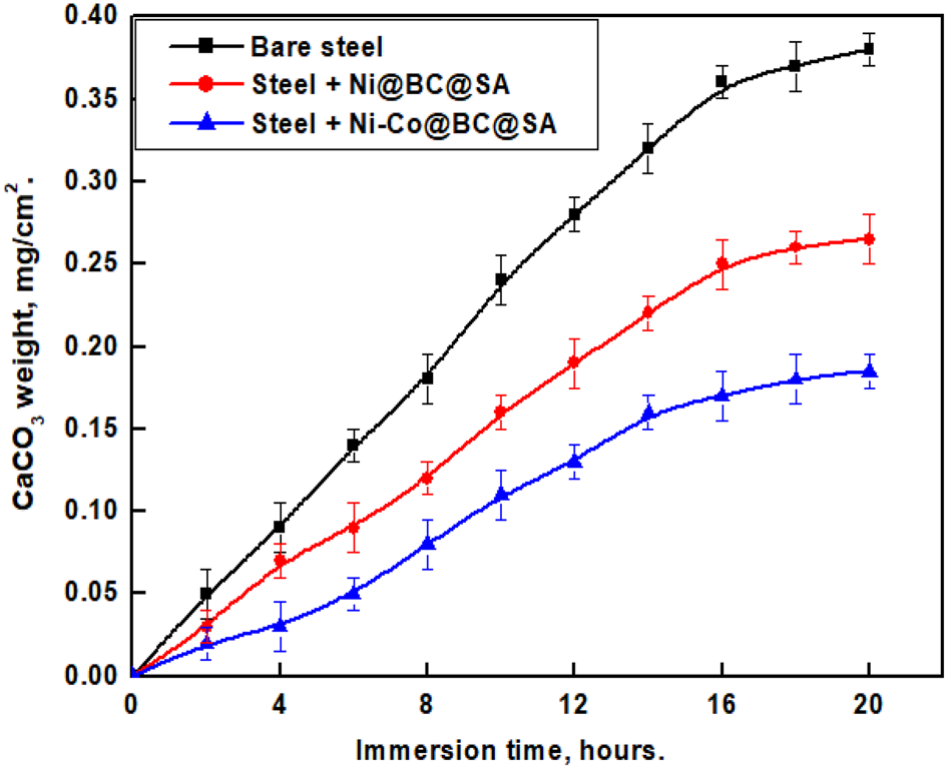
The variation of CaCO_3_ weight on the bare steel and SHP coated steel with immersion time.

The weight gained of CaCO_3_ at the substrate surface is used to test the ability of a given substrate to suppress scale formation and adherence to it. Figure 11 shows the increase in weight of CaCO_3_ (mg/cm^2^) of the bare steel and SHP coated steel every 2 h until 20 h of immersion in a solution of 0.01 M NaHCO_3_ and 0.01 M CaCl_2_ at 60 °C. The Figure shows that the bare steel has a higher weight gain value than steel coated by Ni@BC@SA. So, the prepared SHP coated steel has a lower rate of scale formation due to the intrinsically low surface energy of stearic acid as well as the air pockets between the nano structures^94^. The steel coated by Ni–Co @BC@SA has the lowest weight gain value due to its higher superhydrophobicity, a higher amount of air is trapped between the nanostructures. The weight gain in all cases at low immersion time is linearly increased with immersion time but at high immersion time, a plateau is reached. To calculate the scale inhibition efficiency (% SI), Eq. (4) was used:

4 $$\% {\text{SI }} = \, [ \, \left( {{\text{W}}_{{\text{o}}} - {\text{W}}} \right)/{\text{W}}_{{\text{o}}} ] \times { 1}00,$$

where W_o_ and W are the weight gained by the bare and SHP coated substrates. Table 4 displays the values of W, W_o_, and % SI for the bare and SHP coated steel by Ni @BC@SA and Ni@Co-BC@SA. The values of Wo and W were taken at 20 h of immersion.

**Table 4:** The weight gain and %SI for the bare and SHP coated steel by Ni @BC@SA and Ni@Co-BC@SA.

Sample	Weight gain	%SI
Bare steel	0.380	0.00
Steel + Ni@BC@SA	0.265	30.26
Steel + Ni@Co-BC@SA	0.185	51.32

**Figure 12:**
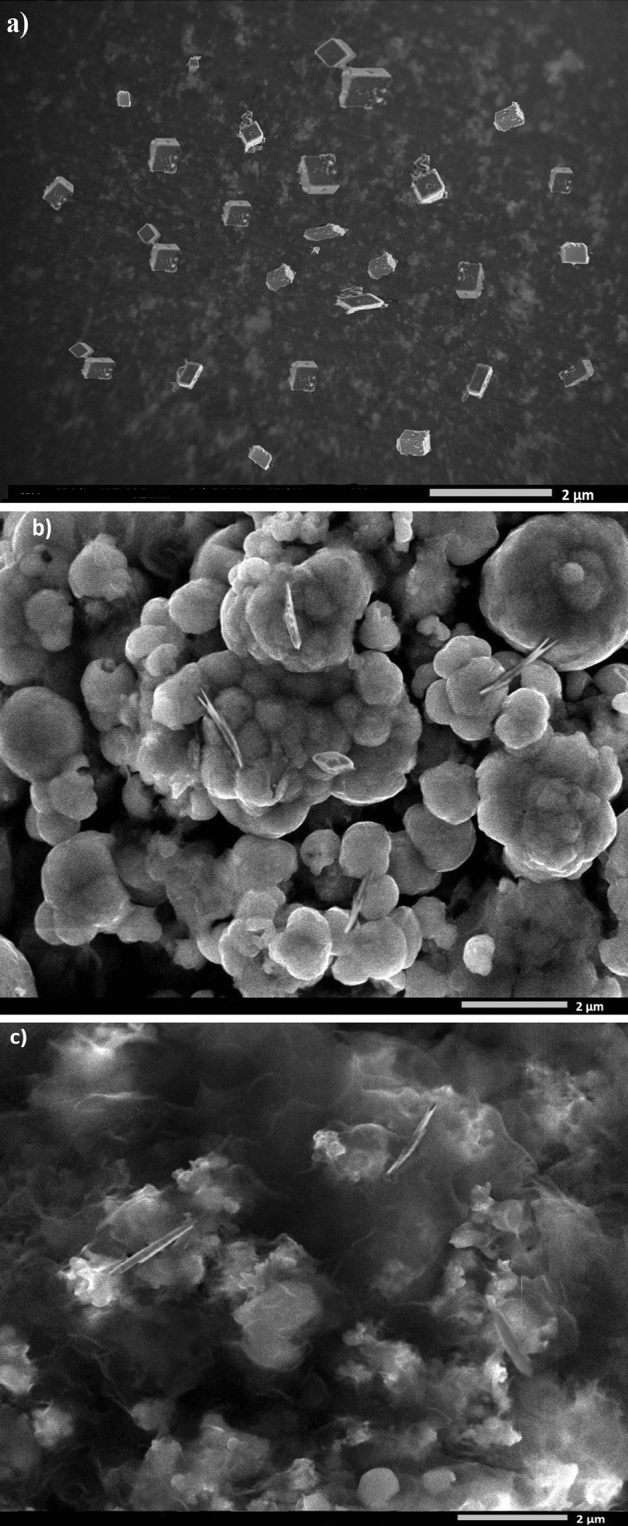
Morphology of scales on the (**a**) bare steel and SHP coated steel by (**b**) Ni@BC@SA, and (**c**) Ni@Co-BC@SA.

SEM was utilized to examine the morphology of CaCO_3_ crystallization on the surface of uncoated and SHP coated steel. According to Fig. 12, rhombic crystals on bare steel mostly depicted the shape of the CaCO_3_ scale, this is consistent with the relatively stable form of ordinary calcite CaCO_3_^95^. However, on the produced SHP coating, the scale's shape clearly changed from rhombic crystals to needle-like structures, which are less stable and adhere to surfaces poorly^95^.

**Figure 13:**
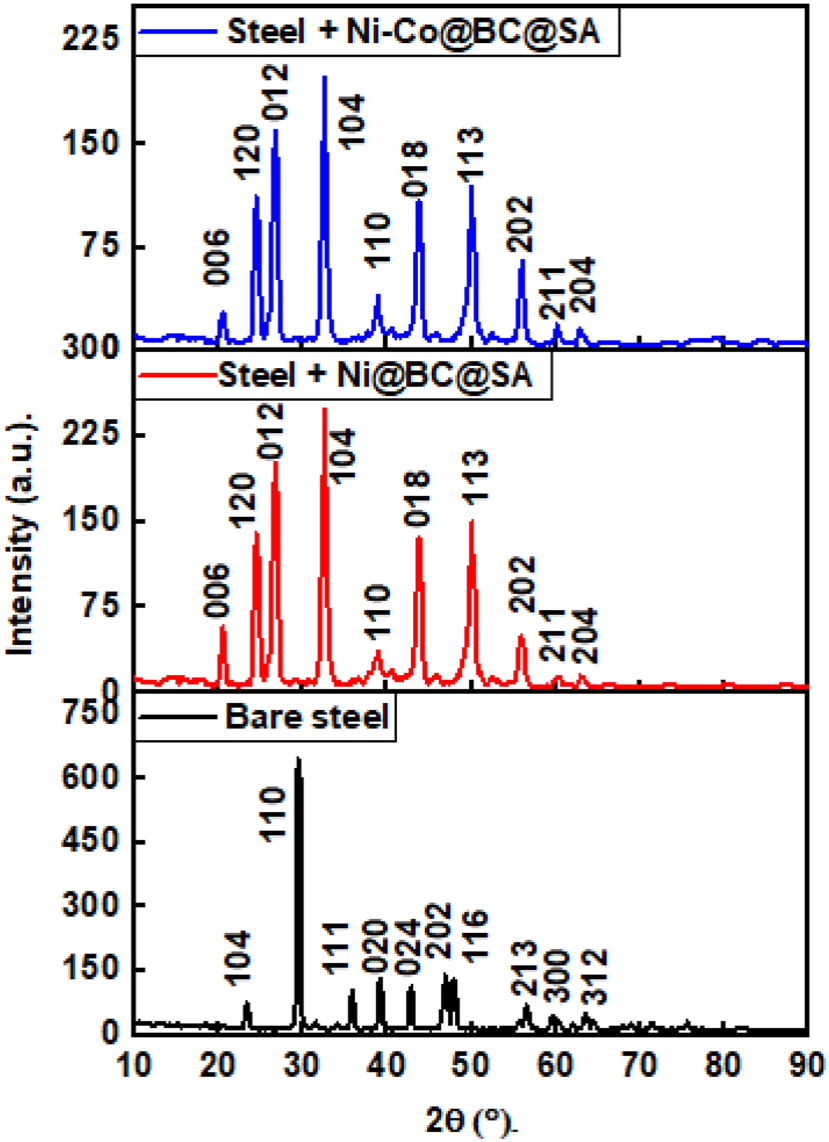
XRD patterns of the scales on the bare steel and SHP coated steel by Ni@BC@SA, and Ni@Co-BC@SA.

The formed scales on the bare steel and SHP coated steel was isolated and their crystal structure was examined using XRD technique, Fig. 13. The results show that the scale formed on the bare steel substrate is composed mainly of calcite, as indicated by the presence of peaks at 2θ equals to 23.5, 29.4, 35.7, 39.2, 42.9, 46.9, 47.8, 56.7, 59.8, and 63.5 degrees. This is consistent with the formation of calcium carbonate scales under typical corrosion conditions. On the other hand, the scale formed on the superhydrophobic coated steel by Ni@BC@SA composed mainly of vaterite, as indicated by the presence of peaks at 2θ equals to 20.3, 24.7, 26.6, 32.6, 38.9, 43.8, 50.1, 55.9, 60.4, and 63.6 degrees. Vaterite is a less stable form of calcium carbonate compared to calcite. The presence of vaterite suggests that the superhydrophobic coating may have influenced the nucleation and growth of the calcium carbonate scales on the surface of the coated steel. The XRD peaks observed for the superhydrophobic coated steel by Ni@Co-BC@SA showed similar peaks to those observed for the superhydrophobic coated steel by Ni@BC@SA but with lower intensity. This suggests that the addition of cobalt to the biochar-based coating did not significantly alter the composition of the calcium carbonate scales formed on the surface of the coated steel but decreases the rate of scale formation^96–98^.

### Ultraviolet stability

**Figure 14:**
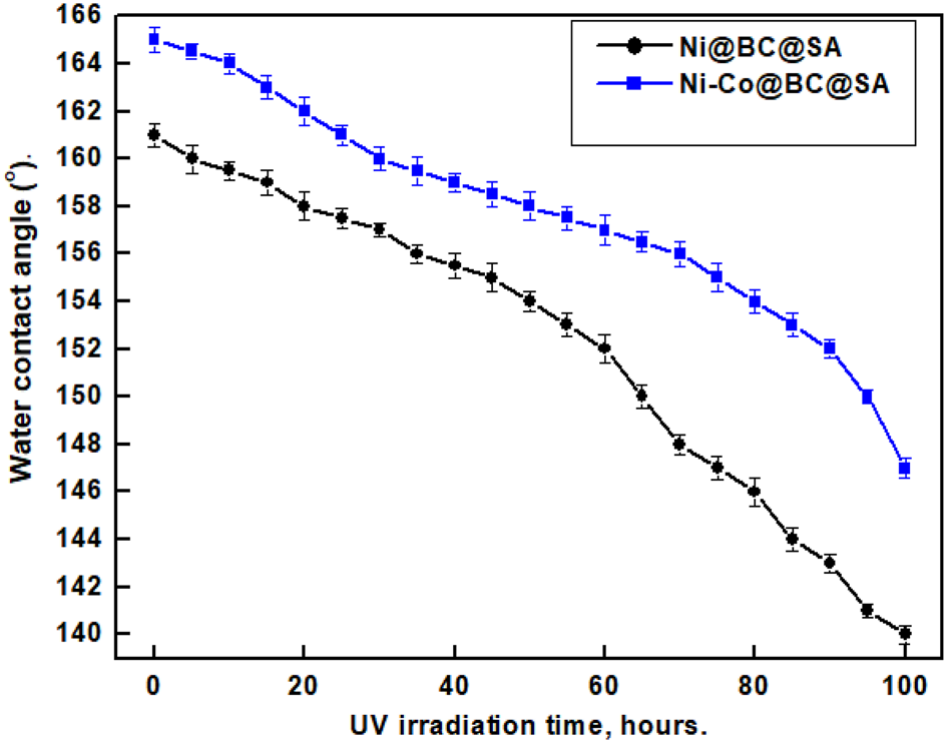
Effect of UV-irradiation time on WCA of the SHP coated-steel by Ni@BC@SA and Ni@Co-BC@SA.

The intrinsic property of the SHP materials themselves mostly determines UV resistance^99^. The manufacture of coatings with UV resilience is a crucial concern for outdoor applications. A SHP surface can have a long UV stability without losing SHP property when a proper choice of materials is made. Figure 14 demonstrates the impact of UV-irradiation time on the WCA of the SHP coated steel by Ni@BC@SA and Ni@Co-BC@SA. The SHP-coated steel by Ni@BC@SA has UV stability of up to 65 h while the steel-coated steel by Ni@Co-BC@SA has UV stability of up to 95 h. The SHP coated steel by Ni@Co-BC@SA has greater UV stability than several previously known values^100–103^.

## Conclusion

1. Biochar was produced using rice straw, an environmentally friendly biomass source, and used to create superhydrophobic coatings of Ni@BC@SA and Ni@Co-BC@SA on a steel substrate. The doping of biochar with cobalt increases the coating superhydrophobicity.
2. The Ni@BC@SA coating retains superhydrophobicity in the pH range of 3–11, whereas the Ni@Co-BC@SA coating retains superhydrophobicity in the pH range of 2–12. Additionally, the created Ni@BC@SA coating demonstrates superhydrophobicity until an abrasion length of 500 mm, while the Ni@Co-BC@SA coating exhibits superhydrophobicity until an abrasion length of 900 mm.
3. According to the PDP findings, the corrosion current density is significantly reduced when steel is coated with a SHP coating, which also results in a significantly reduced corrosion rate. This is further confirmed by the EIS results. The scale inhibition efficiency for coated steel with Ni@BC@SA and Ni@Co-BC@SA is 30.26 and 51.32%, respectively. In terms of UV stability, the coated-steel with Ni@Co-BC@SA remains stable for up to 95 h, whereas the SHP coated-steel with Ni@BC@SA remains stable for up to 65 h.

## Data Availability

The datasets used and/or analyzed during the current study are available from the corresponding author upon reasonable request.
