# Author Correction: Fabrication of biochar-based superhydrophobic coating on steel substrate and its UV resistance, anti-scaling, and corrosion resistance performance

**DOI:** 10.1038/s41598-023-40238-x

**Published:** 2023-08-09

**Authors:** M. E. Mohamed, O. Adel, E. Khamis

**Affiliations:** 1 Chemistry Department, Faculty of Science, Alexandria University, Alexandria, Egypt; 2 Faculty of Advanced Basic Sciences, Alamein International University, Alamein City,, Matrouh Governorate Egypt; 3 Egyptian Russian University, Badr, Egypt

Correction to: *Scientific Reports* 10.1038/s41598-023-36589-0, published online 10 June 2023

The original version of this Article contained an error in the Funding section.

“Open access funding provided by The Science, Technology & Innovation Funding Authority (STDF) in cooperation with The Egyptian Knowledge Bank (EKB). This research did not receive any specific grant from funding agencies in the public, commercial, or not-for-profit sectors.”

now reads:

“Open access funding provided by The Science, Technology & Innovation Funding Authority (STDF) in cooperation with The Egyptian Knowledge Bank (EKB). This paper is based on work supported by a grant from the Science, Technology and Innovation Funding Authority (STDF).”

The original Article has been corrected.

